# Serotonin-Related Gene Polymorphisms and Asymptomatic Neurocognitive Impairment in HIV-Infected Alcohol Abusers

**DOI:** 10.1155/2016/7169172

**Published:** 2016-03-16

**Authors:** Karina Villalba, Jessy G. Dévieux, Rhonda Rosenberg, Jean Lud Cadet

**Affiliations:** ^1^Department of Health Promotion and Disease Prevention, Robert Stempel College of Public Health and Social Work, Florida International University, Miami, FL 33181, USA; ^2^Molecular Neuropsychiatry Research Branch, DHHS/NIH/NIDA Intramural Research Program, Baltimore, MD, USA

## Abstract

HIV-infected individuals continue to experience neurocognitive deterioration despite virologically successful treatments. While the cause remains unclear, evidence suggests that HIV-associated neurocognitive disorders (HAND) may be associated with neurobehavioral dysfunction. Genetic variants have been explored to identify risk markers to determine neuropathogenesis of neurocognitive deterioration. Memory deficits and executive dysfunction are highly prevalent among HIV-infected adults. These conditions can affect their quality of life and HIV risk-taking behaviors. Single nucleotide polymorphisms in the* SLC6A4*,* TPH2*, and* GALM* genes may affect the activity of serotonin and increase the risk of HAND. The present study explored the relationship between* SLC6A4*,* TPH2*, and* GALM* genes and neurocognitive impairment in HIV-infected alcohol abusers. A total of 267 individuals were genotyped for polymorphisms in* SLC6A4* 5-HTTLPR,* TPH2* rs4570625, and* GALM* rs6741892. To assess neurocognitive functions, the Short Category and the Auditory Verbal Learning Tests were used.* TPH2* SNP rs4570625 showed a significant association with executive function in African American males (odds ratio 4.8, 95% CI, 1.5–14.8; *P* = 0.005). Similarly,* GALM* SNP rs6741892 showed an increased risk with African American males (odds ratio 2.4, 95% CI, 1.2–4.9; *P* = 0.02). This study suggests that* TPH2* rs4570625 and* GALM* rs6741892 polymorphisms may be risk factors for HAND.

## 1. Introduction

The development of combination antiretroviral therapy (ART) has mitigated the severity of the human immunodeficiency virus (HIV) epidemic. Therapeutic advances have transformed HIV/AIDS from a life-threatening illness to a chronic condition [1]. Despite substantial improvements in life expectancy and a lower incidence of HIV-associated neurocognitive disorders (HAND), neuropsychological and neurocognitive deficits continue to be highly prevalent [2]. Clinical neurocognitive manifestations of HAND in the ART era differ from the typical AIDS dementia complex [1]. In the pre-ART era, a progressive subcortical dementia with motor and cognitive slowing was common.

However, today more cortical than subcortical involvement is often reported [3, 4]. HAND encompasses a range of cognitive impairments, including slowed processing and deficient memory and attention, decreased executive function, and behavioral changes, such as apathy or lethargy [5]. Although this type of impairment is much more subtle than the classic HIV dementia, it still affects daily function, quality of life, and antiretroviral adherence and can increase HIV risk behaviors [1, 6]. Asymptomatic or minimally symptomatic neurocognitive disorders are more prevalent in individuals in the current ART era than in the pre-ART era [7]. The causes of continuing high rates of HIV-associated neurocognitive disorder in the ART era are uncertain [7]. Comorbid disorders such as aging, coinfection with hepatitis C, and drug abuse may act as moderators of neurocognitive decline [7, 8]. However, there is a need to identify additional risk markers for the development of HAND [9].

Cognitive control processes regulating thought and action are multifaceted functions influenced by heritable genetic factors and environmental influences [10]. The consequences of cognitive impairment are seen by the large range of both neurologic and neuropsychiatric disorders that affect the quality of life [8, 11, 12]. Cognitive impairment is highly heritable, and individual differences in executive function and memory are influenced by genetic variations [13]. Several studies have demonstrated associations between serotonin polymorphisms with sustained attention, memory, and executive function phenotypes in both clinical and nonclinical populations [14–17]. Furthermore, cognitive neuroscience and pharmacology associate dopamine and serotonin as neuromodulators of executive function [10]. Executive function is part of a system that acts in a supervisory capacity in the brain through planning, decision making, and response control for purposeful goal-directed behavior [18]. Memory is a subclass of cognitive function, divided into long-term and short-term (working) memory. Long-term memory is further divided into explicit and implicit memory [19].

Serotonin pathways arise from the dorsal and ventral raphe nuclei to innervate cortical and subcortical brain regions, including the limbic forebrain, basal ganglia, frontal cortices, thalamus, and the hypothalamus [20]. The neurotransmitter serotonin, 5-hydroxytryptamine (5-HT), is implicated in the pathophysiology of several psychological, behavioral, and psychiatric disorders [21]. Molecular genetics studies on 5-HT have shown that single nucleotide polymorphisms in the* TPH2* and* SLC6A4* are associated with impaired memory and executive function [21–23].* SLC6A4* (solute carrier family 6, member 4) encodes the serotonin transporter that affects serotonergic neurotransmission by reuptake of synaptic serotonin, ending neurotransmission [24]. Serotonin reuptake variation is linked to a functional polymorphism in the promoter region of the* SLC6A4* on chromosome 17q11.1–q12 [24]. Polymorphisms within* SLC6A4* have influenced memory regulation, decision making, and response inhibition capabilities [21–23].

The* TPH2* (tryptophan hydroxylase 2) gene encodes a member of the protein-dependent aromatic acid hydroxylase family [15]. It is the rate-limiting enzyme of 5-HT synthesis in the brain, which transforms tryptophan into 5-hydroxytryptophan, the direct precursor of 5-HT [16, 25]. The functional SNP rs4570625 is found within the transcriptional region of* TPH2* on chromosome 12p21.1 [15]. Evidence of* TPH2* variations playing a role in cognition comes from studies implicating* TPH2* in the pathophysiology of ADHD and obsessive compulsive disorder [26–28]. Studies have shown that the homozygous TT genotype in SNP rs4570625 is associated with poorer executive function compared to GG and GT genotypes [15, 22]. These studies showed a compensatory adjustment of deficits in executive control functions [16, 29].

The* GALM* (galactose mutarotase) gene catalyzes the conversion of beta-D-galactose to alpha-D-galactose, which may affect regional neurophysiology, leading to local increases in serotonin release in the brain [30]. A genome-wide association study (GWAS) found a strong association between thalamic region and a coding SNP rs6741892 in* GALM* using the tracer [11C]DASB-BPND, used to measure brain serotonin transporter levels [31].

The study demonstrated that SNP rs6741892 accounted for about 50% of the variance in [11C]DASB-BPND binding potential in the thalamus, especially for the TT genotype [31]. There is accumulated evidence from genetic studies suggesting that genetically determined polymorphisms in serotonin-related genes may amplify differences in cognitive performance measures in individuals with already impaired cognition [32, 33].

The present study explored potential associations with* SLC6A4* 5-HTTLPR,* TPH2* rs4570625, and* GALM* rs6741892 polymorphism and cognitive functions in HIV-infected adults. As described in this section, the existing literature on serotonin genetics and cognition is in HIV-uninfected populations. The present study explored potential associations with* SLC6A4* 5-HTTLPR,* TPH2* rs4570625, and* GALM* rs6741892 polymorphisms and cognitive functions in HIV-infected adults, using a number of cognitive measures reported to be valid and reliable [34, 35].

## 2. Methods

The participants in this study were previously outlined in the study by Villalba et al. [36].

### 2.1. Genotyping

DNA was extracted from whole blood by manual extraction using the QIAamp DNA Mini Kit (Valencia, CA). Genotyping for* TPH2* and* GALM* SNPs was conducted using TaqMan® SNP Genotyping Assays (Foster City, CA) on Bio-Rad CFX96*™* real-time PCR instrument (Hercules CA). Polymerase Chain Reaction (PCR) amplifications were performed by using the Probes Supermix. For the promoter variant called 5-HTTLPR, Bio-Rad CFX Manager software (version 3.0) was used for data acquisition and genotype assignment. The primer sequences used for the 5-HTTLPR amplification were obtained from a previous study [30]. The sequences were as follows: 5′-GCTCTGAATGCCAGCACCTAAC-3′ (forward primer) and AGAGGGACTGAGCTGGACAACCAC-3′ (reverse primer) amplifying 522 bp for the 16-repeat allele and 478 bp for the 14-repeat allele [30]. All genotyping was performed blindly with unknown clinical status or background data on the samples.

### 2.2. Neurocognitive Assessment

All participants were assessed on the same battery of neurocognitive tests and in the same order. Verbal memory was measured with the Auditory Verbal Learning Test (AVLT), using the version World Health Organization/University of California Los Angeles (WHO/UCLA) [37], and executive function was measured by the Short Category Test (SCT) [38]. Alcohol use and other drugs of abuse were also measured using the Timeline Followback (TLFB) and the Alcohol Use Disorders Identification score [39]. There are no agreed criteria to measure HIV-associated neurocognitive disorders. However, the Frascati criteria have been validated and are widely used to classify HIV impairments [3]. The Frascati criteria were used to determine cognitive impairment for asymptomatic neurocognitive impairment (ANI), mild neurocognitive impairment (MND), and HIV-associated dementia (HAD) [40] (Table 1).

#### 2.2.1. Auditory Verbal Learning Test

The AVLT assessment was based on a five-trial presentation of a 15-noun word list (list A) with a presentation rate of one word per second. On completion of trial 5, a single word presentation of a 15-noun word interference list (list B) was presented. The test measured retention, learning, and recognition rates with higher scores representing better episodic memory [41, 42]. This instrument demonstrates high test-retest reliability, with alpha scores ranging from 0.51 to 0.72 [43].

#### 2.2.2. Short Category Test

The SCT assessment consisted of five booklets, one for each subtest, with 20 cards per subtest. All of the cards within each subtest were organized according to a single principle. The test required the individual to formulate an organizing concept for each subtest. The number of errors on each booklet was added to determine impairment with lower scores representing better executive function [38]. Test-retest coefficients range from 0.60 to 0.96, depending upon the severity of impairment in the sample [38].

## 3. Analysis

Statistical analyses were performed using Stata v.11 (StataCorp, College Station, TX). Pearson's *X* ^2^, Student's *t*-test (for means), or median test (for medians) was used to compare characteristics between the participants and nonparticipants. To standardize cognitive measures for this study, standardized* T-scores* were developed by using multiple linear regression methods analyzing the influence of age, sex, education, and ethnicity on each cognitive test score. Each of the five cognitive domains was included as dependent variables: memory (recognition measures form AVLT) executive function (number of errors on the SCT). The continuous predictor was age, and categorical predictors were sex, education, and race/ethnicity. All the predictors in the model were included in each regression, retaining only the variables that significantly contributed to the prediction of cognitive test score. These predictive scores were subtracted from each individual's actual composite score to calculate residual scores. Finally, residual scores were converted to* T-scores* (mean = 50 and SD = 10) which were used to determine cognitive impairment for asymptomatic neurocognitive impairment (ANI). Mild neurocognitive impairment (MND) and HIV-associated dementia (HAD) were not part of the analyses due to small number of participants with MND and HAD. Logistic methods were used to calculate crude and multifactorial (self-reported ethnicity/race, alcohol use severity, viral load, CD4 count, cannabis, and cocaine use) adjusted odds ratios (OR), including 95% confidence intervals (CIs). The ORs, with 95% CIs, were used as a measure of effect size. Test for interaction was performed. Bonferroni method was used for correction for multiple comparisons. All statistical tests were two-tailed, and the threshold for statistical significance was set at *P* < 0.05. Ethnic and gender-specific associations were calculated through stratified analyses. Whenever possible the STREGA reporting guidelines for genetic association studies were used. Genotyping counts were tested for Hardy-Weinberg equilibrium using an exact test. By default, the additive genetic model was used, but due to previous associations in the recessive model for* SCL6A4*, the recessive model was also used.

## 4. Results

A total of 267 HIV-infected alcohol abusers completed the study. Please refer to the study by Villalba et al. [36] for participant characteristics. The Frascati criteria were used to measure neurocognitive impairment. Asymptomatic neurocognitive impairment, greater than one standard deviation below the mean, was observed in 125 (47%) and mild neurocognitive impairment, greater than two standard deviations below the mean, was seen in 11 (4%). HIV-associated dementia was not observed (executive function: mean = 45.2, SD = 10.9; memory: mean = 40.0, SD = 9.1).

Genotyping results, including genotype frequencies, are presented in Tables 2 and 3. All SNPs were in Hardy-Weinberg equilibrium. Analyses yielded significant associations with executive function and* TPH2* and memory and* GALM* genetic polymorphisms.

Whereas 5-HTTLPR polymorphism did not show an association with cognitive flexibility as previously suggested [27], the SNP rs4570625 of* TPH2* gene showed an overall association in the dominant model with impaired executive function (odds ratio = 2.5, 95% CI, 1.1–4.9; *P* = 0.02). Furthermore, the association showed an increased risk in males (odds ratio = 4.0, 95% CI, 1.6–10.5; *P* = 0.007), not in females (*P*_interaction_ = 0.08 for sex). Greater risk was observed in African American males (odds ratio 4.8, 95% CI, 1.514.8; *P* = 0.005). For the SNP rs6741892 of the* GALM* gene, a significant association with impaired memory (odds ratio = 1.9, 95% CI, 1.2–3.1; *P* = 0.006) was observed. The risk again was increased in African American males (odds ratio 2.4, 95% CI, 1.2–4.9; *P* = 0.02). Results from this study showed that the associations between serotonin genes and asymptomatic neurocognitive impairment are male-specific. When stratified by race/ethnicity, results were only significant in African Americans; Caucasians and Hispanics were nonsignificant. The interaction between* GALM* and* THP2* polymorphisms with alcohol use was nonsignificant (*P* = 0.65).

## 5. Discussion

This study provides further evidence for the role of 5-HT in cognition, where functional polymorphisms of two candidate genes in the serotonergic signaling pathway influence executive function and memory. Significant associations were found between* TPH2* SNP rs4570625 and executive dysfunction and* GALM* SNP rs6741892 and impaired memory. Previous studies have shown sex differences in cognition due to dopamine genes interacting with sex and impacting cognition. Similarly, we sought to analyze if serotonin genes were modulated by sex. Thus, stratification by potential effect modifiers (sex and race) showed an even greater effect in African American males but not in females. For the polymorphism 5-HTTLPR, no statistically significant associations were found with neurocognitive measures. Our data suggest that the 5-HTTLPR polymorphism is probably not a risk factor for executive dysfunction and supports previous studies that reported no association between this polymorphism and cognitive decline [44, 45]. However, results are mixed; other studies provided evidence of the influence of 5HTTLPR polymorphism on executive function [14, 46].

The findings of this study indicate that homozygous TT genotype in SNP rs4570625 showed higher error rates measured by the Short Category Test than TG and GG genotypes increasing the risk for executive dysfunction. These findings parallel and extend those of functional imaging and molecular genetic studies suggesting that polymorphism rs4570625 is a risk marker for executive dysfunction [27, 47, 48]. SNP rs4570625 affects the transcription rate of* TPH2*, which may increase the activity of prefrontal cortex [15]. The prefrontal cortex plays a central role in top-down control of many higher-order executive tasks [49–51]. Additional evidence from a functional image study showed a significant association between SNP rs4570625 and increased activity in several prefrontal and parietal sites during updating of working memory [48]. The authors suggested that the effect of SNP rs4570625 was not specific for attention, impulse control, or working memory, rather it seemed to reflect one common basal cognitive process [48]. These studies showed a compensatory adjustment of deficits in executive control functions [15, 16]. Similarly, results in this study are in line with studies suggesting increased prefrontal activity due to serotonin dysregulation affecting executive function [14, 52–54].

Behavioral studies have demonstrated that executive dysfunction (i.e., poor learning) is central to HIV-neurocognitive impairment and most likely affects behaviors, including adherence to antiretroviral medication and unemployment (or underemployment) [7].

Heaton and colleagues found that medically asymptomatic HIV-infected adults with executive dysfunction were twice as likely to be unemployed and perceived greater vocational difficulties than their unimpaired counterparts [55]. Similarly, another study showed that, for recently diagnosed individuals, the key predictors for finding employment were learning and memory [56].

This study is the first to analyze the functionality of the* GALM* SNP rs6741892 in relation to 5-HT transporter. Thus, a significant association between rs6741892 and impaired memory measured by Auditory Verbal Learning Test was found in HIV-infected adults.

The Auditory Verbal Learning Test was used because of the repeated presentations of words and their successive testing at various time intervals which allowed for the analysis of different memory learning processes such as acquisition, retention, retrieval, and interference [41]. The thalamus plays a significant role in regulating higher level brain activity [57]. Several functional imaging studies showed a relationship between memory processes and the thalamus [58]. Results in this study showed an association with explicit memory and are consistent with neuroimaging reports of compromised thalamus and associated memory structures [57, 58]. Explicit memory is correlated with limited use of higher level encoding strategies, such as semantic clustering and strategic retrieval. This can lead to issues involving medication nonadherence and problematic work-related issues in HIV-infected adults [56, 59]. This study has several limitations. First, there was relatively low frequency of homozygous TT genotype of the* TPH2* SNP. However, it should be noted that there is relatively low occurrence of TT genotype within the general population. In fact, compared to previous studies, the current study included a rather high proportion of homozygous TT genotype carriers compared to others [15, 60]. Second, because of the low power of the study to detect smaller effect sizes, some important associations may not have emerged as statistically significant. Multiple comparisons were necessary due to the exploratory nature of the study, including the analysis of the SNP functionality in the* GALM* gene, as well as the use of all three genetic models. These results should be viewed with caution and should be replicated before a definitive conclusion can be drawn. In general the additive model is used to assess statistical associations of SNPs. While the additive model has sufficient power to detect associations in most situations, there may be occasions where statistical significance is not found, when, in fact, there is an association. Consequently, a strength in this study was the use of multiple genetic models to determine associations that may remain undetectable by the exclusive use of the additive model. Third, due to the vast number of HIV antiretroviral drugs used by study participants, we did not adjust for HIV medication type. Since certain HIV antiretroviral drugs may also affect cognition, this may potentially confound the results. Fourth, two main approaches are used to approximate individual ancestry in association with studies, self-reported race, and ancestry informative markers. We did not use ancestry informative markers due to DNA requirements. Instead, we used self-reported ancestry that may capture common environmental influences as well as ancestral background. However, self-identified racial categories may not always consistently predict ancestral population clusters. Finally, since this was a cross-sectional study stemming from a behavioral intervention trial of HIV-infected subjects, we did not have a healthy control group. Although we adjusted for alcohol and drug use, the results may not adequately explain whether impairments in memory and executive function were correlated with the presence of SNPs* TPH2* rs4570625 and* GALM* rs6741892 or mediated by HIV and alcohol/drug use. Alternatively, these results can serve as an initial point for future research in cognitive phenotypes for HAND in adults. Molecular genetics, as applied in the present study, offers further analytic insight beyond behavioral assessment and neuroimaging and may present a reasonable instrument for the dissociation of different executive control processes.

This study may pave the way for future research integrating the examination of genetic factors in behavioral prevention interventions with HIV-infected populations. Future studies are needed to further identify specific neurocognitive aspects that are especially relevant to HIV-associated neurocognitive disorders. Studies that incorporate genetic factors in combination with neurocognitive testing would benefit from also including the effects of genetic factors on cognitive functioning in healthy individuals since gene by disorder interactions might be expected. Furthermore, it would be beneficial to investigate haplotypes rather than genotypes in studies on cognitive performance in HAND. Since most of the polymorphisms have a small relative effect on cognition, a larger sample is optimal to detect an effect. In addition to the genes analyzed in this study, other genes related to cognitive function should be included. The available findings provide preliminary information for identifying targets for cognitive rehabilitation and other behavioral interventions.

## 6. Conclusion

The current study was the first to explore the relationship between serotonin-related polymorphisms and asymptomatic neurocognitive impairment in a sample of HIV-infected adults. The results showed a significant association between* TPH2* rs4570625 and individual differences in executive function and* GALM* rs6741892 with memory. The two associations were male-specific. The present study validates previous results pointing to genetic influences on executive function and memory. Moreover, a significant association between SNP rs6741892 and memory was demonstrated, which may imply SNP rs6741892 as a functional polymorphism in the* GALM* gene.

## Figures and Tables

**Table 1:** Categories of HIV-associated neurocognitive disorder according to Frascati criteria.

	Neurocognitive status^**∗**^	Functional status
Asymptomatic neurocognitive impairment	1 SD below the mean in 2 cognitive domains	No impairment in activities of daily living
Mild neurocognitive impairment or disorder	1 SD below the mean in 2 cognitive domains	Impairment in activities of daily living
HIV-associated dementia	2 SD below the mean in 2 cognitive domains	Notable impairment in activities of daily living

SD: standard deviation.

^*∗*^Neurocognitive testing should include an assessment of at least five domains, including attention-information processing, language, abstraction-executive, complex perceptual motor skills, memory (including learning and recall), simple motor skills, or sensory, perceptual skills.

**Table 2:** Genotype frequencies and cognitive scores for AVLT and SCT tests.

Chr.	Position	Gene	Variant	Minor allele	A/A	A/B	B/B	MAF	Cognitive *T-scores*
									Mean (SD)
12	12:71938143	*TPH2*	rs4570625	T	93	120	47	0.18	40.6 (15.1)
2	2:38689828	*GALM*	rs6741892	T	90	114	60	0.22	47.4 (9.8)

**Table 3:** *TPH2 *and *GALM *associations with cognitive impairment stratified by sex and race/ethnicity (ORs and 95% CIs).

Gene	Variant	Domain	OR allele (95% CI)	*P* value	OR sex (95% CI)	*P* value	OR race (95% CI)	*P* value	OR race × sex (95% CI)	*P* value
*TPH2*	rs4570625	Executive function	2.5 (1.1 to 4.9)	*P* = 0.02	4.0 (1.6 to 10.5)	*P* = 0.007	3.3 (1.4 to 7.6)	*P* = 0.007	4.8 (1.5 to 14.8)	*P* = 0.005
*GALM*	rs6741892	Memory	1.9 (1.2 to 3.1)	*P* = 0.006	2.3 (1.2 to 4.2)	*P* = 0.009	1.9 (1.1 to 3.6)	*P* = 0.02	2.4 (1.2 to 4.9)	*P* = 0.02

OR stratified by sex, OR stratified by race, OR stratified by sex and race.
